# Study of Wire-Cut Electro-Discharge Machining of Heat-Resistant Nickel Alloys

**DOI:** 10.3390/ma16206743

**Published:** 2023-10-18

**Authors:** Timur Rizovich Ablyaz, Evgeny Sergeevich Shlykov, Karim Ravilevich Muratov, Sarabjeet Singh Sidhu, Dmitry Mikhailovich, Khairulin Vadim Takhirovich

**Affiliations:** 1 Mechnical Engineering Department, Perm National Research Polytechnic University, Perm 614990, Russia; kruspert@mail.ru (E.S.S.); karimur_80@mail.ru (K.R.M.); 2 Mechanical Engineering Department, Sardar Beant Singh State University, Gurdaspur 143521, Punjab, India; 3 Institute of Technical Chemistry of the Ural Branch, RAS, Perm 614990, Russia; dkiselkov@yandex.ru; 4 UEC Perm Motors, Perm 614010, Russia; khairulin-vt@mail.ru

**Keywords:** wire-cut EDM, high-temperature nickel alloys, surface roughness, cutting width

## Abstract

This paper presents an analysis and theoretical model for assessing the quality and accuracy of wire-cut electro-discharge machining (WEDM) of products made from novel heat-resistant nickel alloys such as CrNi56KVMTYB. It is observed that WEDM processing of Ni alloy led to high surface roughness for the thick specimens, and electrical parameters such as pulse duration for the selected range depict an insignificant role in the value of surface roughness. On the other hand, the cut width of the machined surface decreases as the pulse duration increases, while the cut width is elevated for thick workpieces. Secondary discharges developed in WEDM have negative effects that cause sludge adhering and deterioration in the quality and productivity of processing. The regression model is developed to predict the surface roughness and cut width of machined surfaces, which holds significant importance in modern engineering. The workpiece is examined for surface integrity and material deposition. It is observed that an increase in the height of the specimen leads to the occurrence of secondary discharges, which in turn results in the formation of cracks on the surfaces of high-temperature nickel alloys. These cracks have a detrimental effect on the performance of critical products made from next-generation heat-resistant nickel alloys.

## 1. Introduction

In the context of the industrial revolution, stringent demands are being placed on the precision, quality, and dependability of manufactured goods. Over the past few decades, the need to enhance the reliability, environmental sustainability, specific thrust, and fuel efficiency of power plants has gained considerable importance. The adoption of heat-resistant materials exhibiting exceptional mechanical and physicochemical attributes stands as a critical factor in the aerospace sector. The utilization of heat-resistant nickel (Ni) alloys emerges as a solution to address these challenges. By employing these materials, the wear resistance, heat resilience, and operational characteristics of the resultant products have been significantly augmented.

These advanced materials facilitate the creation of products capable of withstanding high-temperature stresses. Notable examples include heat-resistant nickel alloys such as CrNi56KVMTYB and CrNi50VMTYB, which exhibit remarkable endurance under elevated temperatures. These alloys find practical applications in diverse industrial settings, including jet engines and steam turbines [1,2]. Distinguished by their composition—comprising 10–12% chromium, 50–56% nickel, a ferrous alloy, 5–6% molybdenum, 3% titanium, and 14–16% cobalt—these novel alloys are engineered to function under exceedingly harsh and challenging conditions.

A paramount criterion for alloys destined for use in gas turbine engine blades revolves around their capacity to endure high-temperature conditions within the range of 950–1100 °C, while simultaneously withstanding stress levels ranging from 150 to 250 MPa. The spectrum of applications for heat-resistant alloys continues to expand each year, driven by their exceptional attributes, including resistance to extreme temperatures, formidable thermal strength, minimal susceptibility to creep, corrosion resistance, and adaptability to hostile environments [3,4,5]. Traditional machining methods, such as turning or milling, are commonly employed for working with alloys. However, processing heat-resistant materials using these conventional methods presents challenges due to the high tool wear. For instance, when machining high-temperature nickel alloys, significant pressure is exerted on the tool, leading to the release of a substantial amount of heat [6]. The elevated temperatures adversely affect the lifespan of cutting tools and prolong processing time, thereby increasing the overall manufacturing costs of high-quality products. Researchers have found that the friction between the particles falling off the tool matrix and the hard carbon particles contained in the nickel-based superalloy is the main cause of abrasive wear when machining nickel-based superalloys with various conventional tool materials. Moreover, since the workpiece material flows to both sides during machining, the hardened burrs formed on the workpiece surface also cause damage to the tool after the end of the previous machining process, resulting in abrasive wear. The occurrence of abrasive wear is usually marked by the depths of the grooves on the rake face and flank face of the tool and the depth of the notch at the contact point between the tool and the workpiece [7].

Effectively processing these materials can be achieved through techniques based on electrodischarge energy. Among these methods, electrical discharge machining (EDM) is a popular choice and finds application in various tool rooms. Some researchers have explored the machining of Ni alloys using non-traditional techniques and have found wire-cut electrical discharge machining (WEDM) to be an effective method. WEDM is particularly suitable for processing complex profile products made from heat-resistant nickel alloys, providing both accuracy and surface quality. The material removal from the craters depends on various EDM process parameters, including discharge current (I, A), discharge voltage (U, V), pulse duration (Ton), pulse off time (Toff), workpiece material, and electrode tool material [8,9,10,11,12,13].

Daneshmand [14] investigated WEDM responses, specifically the material removal rate (MRR) and surface roughness (SR), of the NiTiNOL60 alloy. This study identified pulse duration and current as significant factors influencing MRR and SR. Similarly, the impact of cutting parameters on WEDM for NiTiNOL60 [15] highlighted that a higher pulse time resulted in numerous surface craters due to the generation of a large number of sparks during WEDM, with rapid cooling leading to layer formation on the machined alloy. As a result, the solidified layer exhibited higher microhardness compared to the base part. Sharma [16] conducted optimization studies on WEDM for porous Ni40Ti60 alloy, establishing relationships between peak current, pulse time, pause time, and servo voltage using response surface methodology (RSM) with a central composite rotatable design. They concluded that a single-cut operation is sufficient for WEDM of NiTi alloys.

It may be possible to process these superalloys using non-convectional manufacturing techniques. Among these techniques, WEDM is highly suited for fabricating gas turbine blades because it allows for greater freedom to cut complex shapes with excellent precision. In this process, the cutting force to remove material is absent, thus reducing the residual stresses in machined workpieces [17].

Much of the current research focuses on processing non-heat-resistant complex nickel alloys, which find applications in medicine and other domestic industries. However, the processing of new-generation high-temperature nickel alloys remains relatively understudied. WEDM processing of these new materials necessitates the prediction of the interelectrode gap, surface quality, and other parameters. Typically, pilot experimentation is employed to ensure machining accuracy in manufacturing enterprises. However, this testing phase elongates the introduction time of new technology for the latest materials. To expedite this testing period, processes such as theoretical modeling, factorial experiments, and regression analysis [18,19,20,21,22,23,24] are utilized to streamline technology implementation time.

According to the literature, the machining of CrNi56KVMTYB has not been explored extensively. Similarly, the application of optimization in CrNi56KVMTYB machining, specifically about surface roughness and kerf width, is not examined. Hence, investigating the machining performance of WEDM on Nimonic (heat-resistant nickel alloys) alloys is highly required in the current scenario. The novelty of this research lies in investigating the effects of input process parameters on the properties of Nimonic alloy materials using the WEDM process. Understanding the influence of machining factors on machining performance is crucial for modern industry, particularly in achieving a higher material removal rate, excellent dimensional accuracy, and superior surface finish. Therefore, the main objective of this study is to investigate the machining performance, including the material removal rate, surface roughness, and kerf width of Nimonic alloy using the wire electric discharge machining process.

The focus of the present study is to identify the significant process parameters and develop a theoretical model that establishes the relationship between WEDM parameters and the quality of products obtained from heat-resistant nickel alloys. This research endeavors to develop empirical models that elucidate the connection between surface roughness and cut width on machined surfaces.

## 2. Material and Methods

### Material

The material chosen for experimentation was the heat-resistant nickel alloy CrNi56KVMTYB (from JSC Vse-rossiysky Institute of Light Alloys, Moscow, Russia). The blanks used were of sizes 15 × 10 × 1.5 mm and 10 × 10 × 1.5 mm. The machining process was carried out using a wire-cut EDM machine called Electronica EcoCut (manufactured by Electronica Machine Tools, Pune, India). An EDM wire with a diameter of 0.25 mm from Berkenhoff GmbH (Herborn, Germany) was used as the tool electrode, and distilled water was employed as the working fluid. The surface roughness of the machined surfaces was measured using a profilometer (Perthometer S2, Mahr GmbH, Goettingen, Germany), with a base length of 0.8 mm. The response parameters analyzed were average roughness (Ra) and surface morphology. To study the surface characteristics of the specimens, a scanning electron microscope (Hitachi S-3400N, Hitachi—Science & Technology, Tokyo, Japan) was used in a backscattered electron trial.

The evaluation of surface roughness was performed using a 3D image model on a laser scanning microscope (Olympus Corporation, Tokyo, Japan) coupled with the 3D Roughness Reconstruction software module (Olympus Corporation, Tokyo, Japan). Optical sections obtained in *X*-*Y*-*Z* scanning of specific surface areas were used to construct 3D surface models. Scanning was conducted at magnifications of ×200 and ×1000, with a scanning step of 2 μm along the *Z* axis. A laser with a wavelength of 405 nm served as the light source for the scanning process. An Olympus GX 51 light microscope (Olympus Corporation, Tokyo, Japan) was used to assess texturing outcomes and measure cutting width.

**Figure 1:**
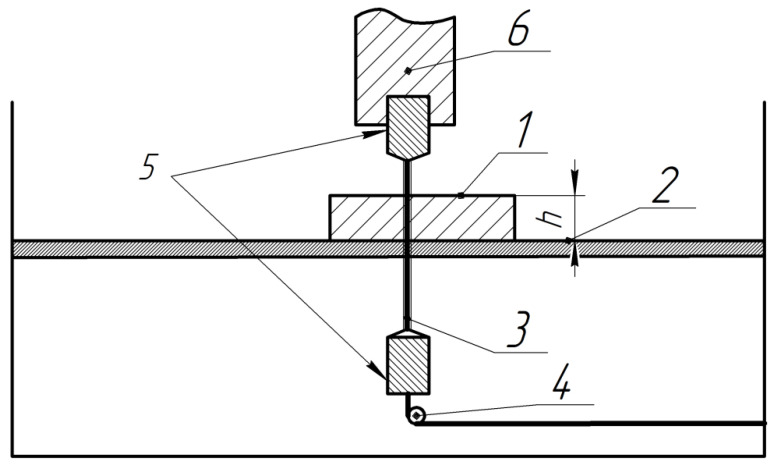
Schematic diagram of WEDM: 1—workpiece, height h, mm, 2—working table, 3—wire electrode, 4—tension roller, 5—die, 6—drive.

For the collection of experimental data, a factorial experiment was conducted. The input parameters selected were pulse on time (Ton, µs), pulse off time (Toff, µs), and workpiece height (h, mm). The level of parameters is indicated in Table 1. The schematic diagram of the WEDM process is depicted in Figure 1.

The cut width Y (µm) and surface roughness Ra (µm) were the output parameters. The experimental design matrix is shown in Table 2.

The experimentation is repeated thrice and, based on the results of the experiments, the arithmetic mean value of the output parameter is obtained.

## 3. Results and Discussion

The output responses for surface roughness Ra (µm) and cut width Y (µm) are shown in the 2nd last and the last column of Table 2, respectively.

### 3.1. Analysis of Variance for Surface Roughness

The results for the surface roughness were analyzed using analysis of variance (ANOVA). A summary of the ANOVA is provided in Table 3. Comparing the data F-values with the F critical at a confidence level of 95%, the significant factors were identified. The higher the F-value, the higher the effect of the parameter was on the response.

**Figure 2:**
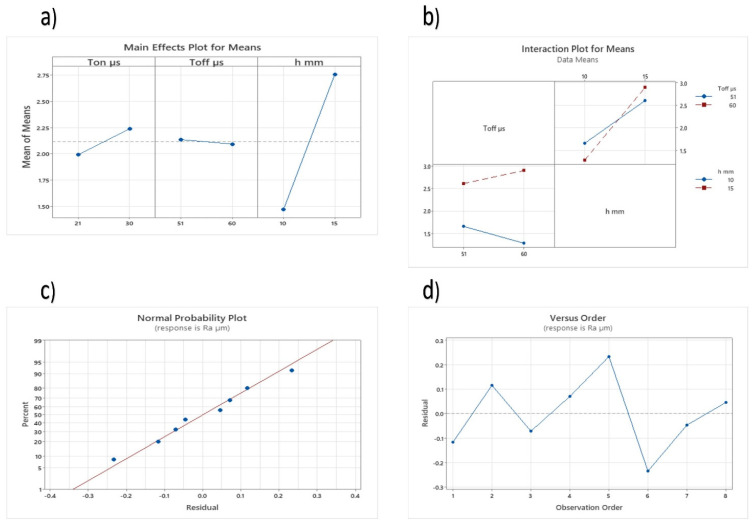
Surface roughness: (**a**) main effect plot, (**b**) interaction plot of means, (**c**) probability plot of trial, and (**d**) representation of observed value and the residual values.

Experimental outcomes were analyzed with the help of Minitab-19, and the evaluation of surface roughness for the WEDM parameters was revealed through analysis of variance (ANOVA). It was also evident that the higher thickness (h, mm) of the specimen has been reported as the most dominant factor influencing the surface roughness (Table 3). From Table 3, it can be observed that there is an interaction between pulse off time (Toff) and specimen thickness, which affects the surface roughness of the specimen. The mean effect plot and interaction plot based on the input variables and output response are shown in Figure 2.

The general regression analysis was used to develop a nonlinear trial from the experimental data. From the results obtained, the final regression trials for machining under the given condition are given by Equation (1) for surface roughness:

The final trial is represented as

Ra = 8.72 + 0.0275 Ton − 0.1894 Toff − 0.564 h + 0.01478 Toff × h(1)

R-sq = 96.02% and R-sq(adj) = 90.71%

The adequacy of the predicted trial is examined by a normal probability plot (Figure 2c) and a residual plot (Figure 2d). The plots prove that the developed trial is appropriate to predict the surface roughness of selected Ni-alloy machined via the WEDM process.

### 3.2. Analysis of Variance for Cut Width

The results for the cut width were analyzed using analysis of variance (ANOVA). A summary of the ANOVA is provided in Table 4.

**Figure 3:**
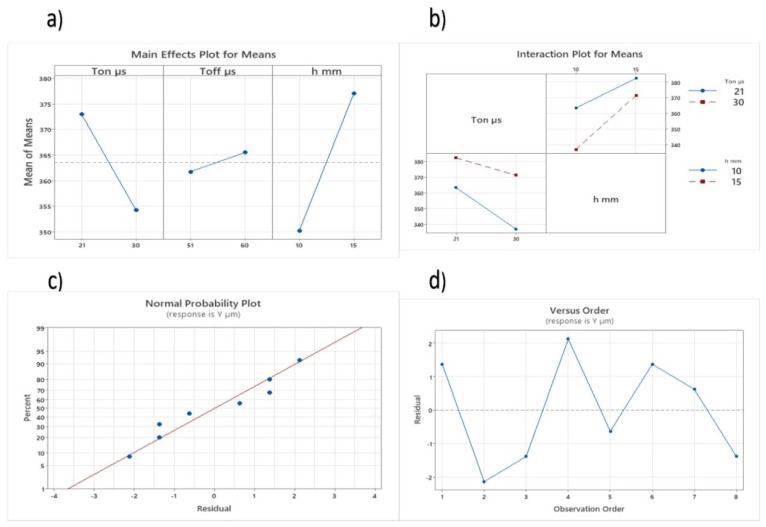
Cut width: (**a**) main effect plot for cut width, (**b**) interaction plot of means for cut width, (**c**) probability plot of trial for cut width, and (**d**) observed value and plot of the residual values for cut width.

The ANOVA for the cut width shows that pulse on time and workpiece width significantly affected the residual stresses. It is also observed that there is a strong interaction between these parameters (i.e., Ton and h). It can be seen from the results that pulse off time showed no effect on the cut width of the workpiece. The main effect plots and interaction plot of the response are given in Figure 3. Figure 3 shows the variation in the response plotted on the *y*-axis with the change in parameter settings. This graph shows that the cut width decreases with an increase in Ton time and increases with an increase in the thickness of the sample.

The regression analysis was used to develop atrial from the experimental data of cut width. From the results obtained, the final regression trials obtained is given by Equation (2):

The final trial is represented as

Y = 436.5 − 6.389 Ton + 0.417 Toff − 3.43 h + 0.3444 Ton × h(2)

R-sq = 99.24% and R-sq(adj) = 98.24%

The adequacy of the predicted trial is depicted by R-squared (R-sq) values, and graphically represented by a normal probability plot (Figure 3c) as well as a residual plot (Figure 3d). These plots demonstrate that the developed trial is suitable for forecasting the cut width of Ni-alloy machined via the WEDM process.

### 3.3. Microstructure Analysis for Surface Roughness

**Figure 4:**
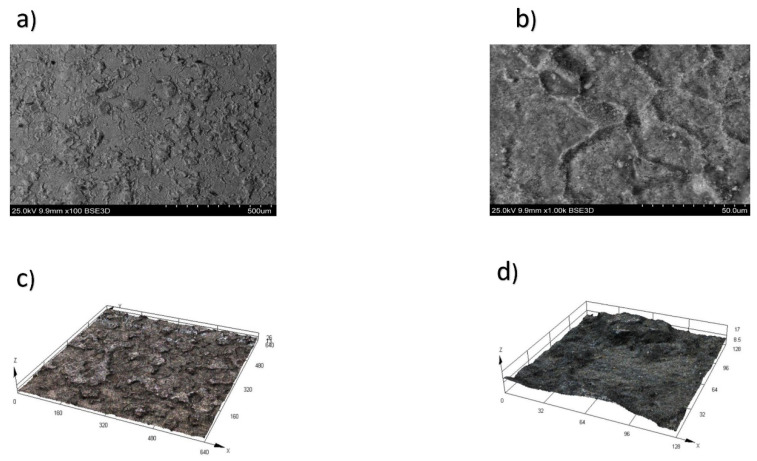
Surface of the CrNi56KVMTYB alloy after WEDM in trial 3. (**a**) Magnification ×100, (**b**) magnification ×1000, (**c**) 3D trial of the topography magnification ×100, and (**d**) 3D trial of the topography magnification ×1000. This revealed a reduction in the formation of microcraters, leading to a more uniform dispersion of molten material across the surface. Additionally, Figure 4c,d demonstrate the absence of molten material in the form of holes, ridges, depressions, or sharp influxes. Instead, the surface exhibits a smooth and consistent morphology.

These dependencies have been established for the first time for the heat-resistant nickel alloy CrNi56KVMTYB. As this alloy is a newly developed material in Russia, there were no previous findings regarding the formation of the roughness parameter for it. The surfaces of the alloy during WEDM in trial 3 at a height of h = 10 mm, with a pulse off time Toff = 60 µs and an on time Ton = 21 µs, are shown in Figure 4a (100× magnification) and Figure 4b (1000× magnification). The surface integrity of the surface layer stands as one of the primary criteria for assessing the quality of processed parts. An analysis of micrographs (depicted in Figure 4) of the heat-resistant nickel alloy after Wire Electrical Discharge Machining (WEDM) enables us to draw accurate conclusions. Three-dimensional (3D) trials depicting the topography of the treated surface following WEDM in trial 3, magnified at scales of ×100 (Figure 4a) and ×1000 (Figure 4c), are illustrated in Figure 4d.

**Figure 5:**
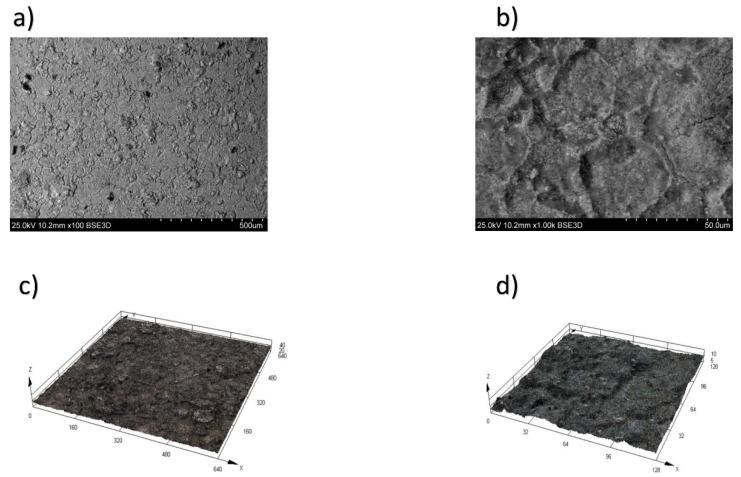
The surface of the KhN56KVMTYUB alloy after WEDM in trial 5. (**a**) Magnification ×100, (**b**) magnification ×1000, (**c**) 3D trial of the topography magnification ×100, and (**d**) 3D trial of the topography magnification ×1000.

The surfaces of the alloy during WEDM in trial 4, with a height of h = 10 mm, pulse off time Toff = 60 μs, and on time Ton = 30 μs, are displayed at magnifications of ×100 (Figure 5a) and ×1000 (Figure 5b) times in Figure 5a and 5b, respectively. The melting zone of the alloy became evident at elevated pulse energy (Ton), leading to the formation of cavities and variously shaped influxes. This consequently resulted in heightened surface roughness within the machined zone. The material’s surface layer experienced substantial thermal shocks during WEDM, leading to the development of secondary depositions on its surface.

Three-dimensional (3D) trials illustrating the topography of the treated alloy surface during WEDM in trial 4, magnified at scales of ×100 (Figure 5c) and ×1000 (Figure 5d), are depicted in Figure 5.

An escalation in pulse energy leads to an augmentation in the roughness parameter and triggers the onset of cracks. The emergence of cracks arises from the swift elevation of the workpiece surface temperature beyond 5000 °C, succeeded by rapid cooling to the temperature of the dielectric liquid (20 °C). Consequently, metal cracking transpires due to the influence of thermal stresses, where the presence of depressions and holes assumes a significant role as stress concentrators.

**Figure 6:**
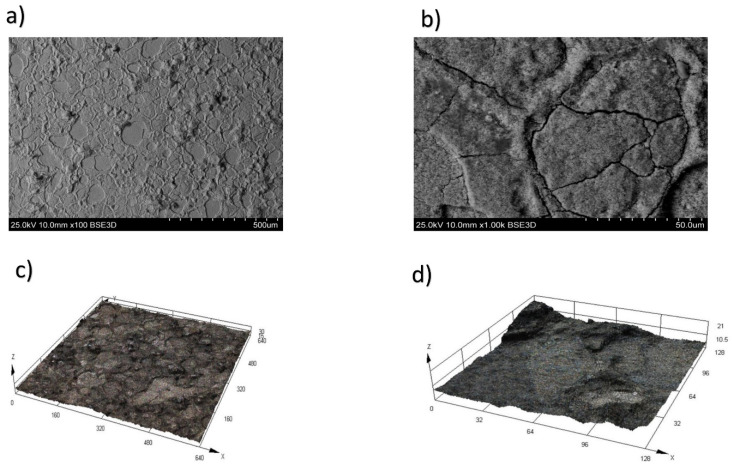
The surface of the CrNi56KVMTYB B alloy after WEDM in trial 7. (**a**) Magnification ×100, (**b**) magnification ×1000, (**c**) 3D trial of the topography magnification ×100, (**d**) 3D trial of the topography magnification ×1000.

The surface of the alloy during WEDM in trial 7, with a height of h = 15 mm, pulse off time Toff = 60 µs, and on time Ton = 21 µs, is depicted at magnifications of ×100 (Figure 6a) and ×1000 (Figure 6b) in Figure 6a and 6b, respectively.

Deposition and the consequent formation of cracks resulting from WEDM become evident in trial 7, thereby deteriorating the surface and worsening the performance characteristics of the products. The mechanism of WEDM and, correspondingly, the formation of cracks on the alloy surface, can be delineated. Initially, the customary process of electrolysis takes place. This process persists until a threshold current is reached. At this specific current level, initial bubbles form, followed by subsequent secondary discharges. These discharges coincide with the generation of bubbles on the cathode surface. The augmentation in gas formation and bubble appearance is attributed to the escalation of the local temperature adjacent to the cathode. The proliferation of numerous small bubbles on the cathode intensifies the occurrence of secondary discharges, as illustrated in Figure 6.

Three-dimensional (3D) trials representing the topography of the treated alloy surface during WEDM in trial 7, magnified at scales of 200 (Figure 6c) and 1000 (Figure 6d) times, are displayed in Figure 6.

The final stage was characterized by the phenomena of cavitation, as well as plasma channel (with a lifetime of 5 μs). This plasma formed due to the increase in high-frequency discharges near the cathode. The bubbles formed at the gas–vapor layer begin to collapse due to the balancing of the internal and external pressure and increase in the local temperature. This temperature leads to a large cavitation shock, which is approximately 1010 MPa. It also leads to a further active increase in the surface roughness parameter and the growth of cracks during WEDM of a heat-resistant nickel alloy.

**Figure 7:**
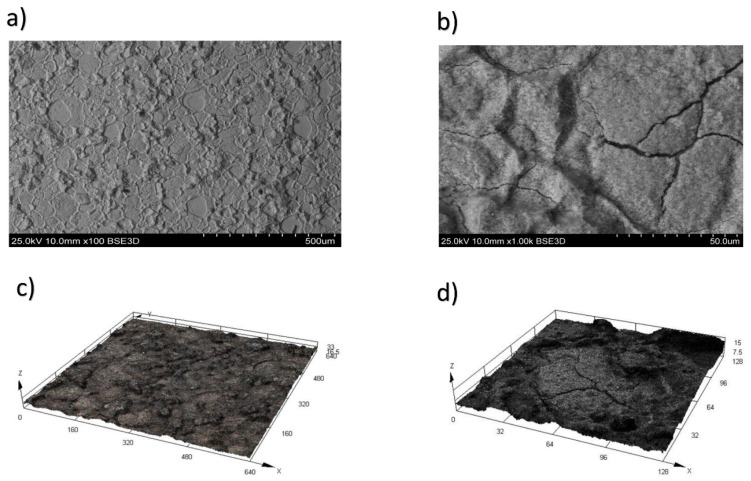
Surface of the CrNi56KVMTYB after WEDM in trial 7. (**a**) Magnification ×100, (**b**) magnification ×1000, (**c**) 3D trial of the topography magnification ×100, and (**d**) 3D trial of the topography magnification ×1000.

In Figure 7a,b, the surface of the sample during WEDM in trial 8 at a height h = 15 mm, the pulse off time (Toff) = 60 μs, and the on time (Ton) = 21 μs at magnifications of ×100 (Figure 7a) and ×1000 (Figure 7b) times are shown. In Figure 7, WEDM at trial 8 and processing height h = 15 mm is presented. In this case, the surface layer of the workpiece material was subjected to intense thermal stress during WEDM. The value of the Ra roughness parameter and the size and intensity of cracks increase with increasing pulse energy and workpiece height.

3D trials of the topography of the treated surface of the alloy during WEDM in trial 7 at magnifications of ×100 (Figure 6a) and ×1000 (Figure 6b) times are shown in Figure 6.

An intensified concentration of energy on the processing surface takes place when the height of the workpiece being processed is elevated. This leads to an adverse effect, resulting in the formation of cracks on the surface. These cracks exert a detrimental influence on the operational properties of critical products.

#### Cut width Micrograph Analysis

**Figure 8:**
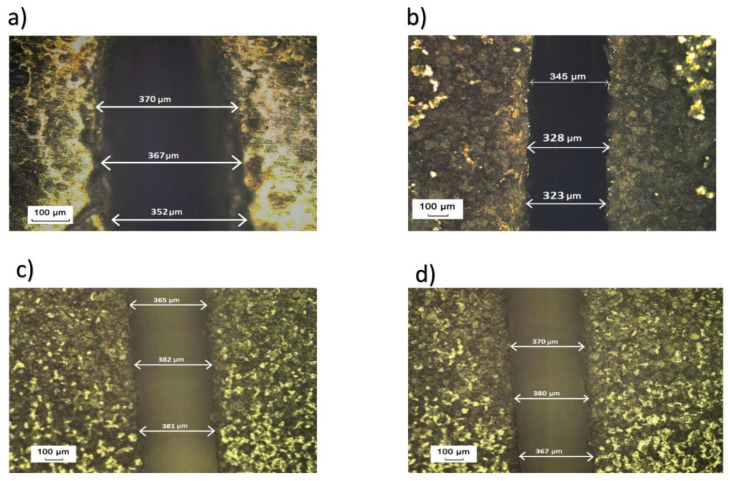
Cut width at ×100: (**a**) trial 3, (**b**) trial 4, (**c**) trial 7, and (**d**) trial 8.

Measurements of the cut width for the workpieces with thicknesses of 10 mm and 15 mm, respectively, obtained in trials 3, 4, 7, and 8, with a magnification of ×100, are presented in Figure 8.

It has been established that with increasing the value of pulse on time, the cut width decreases; however, the value of the cut width changes with an increase in the workpiece height.

In the future, it will be critical to develop improved theoretical models for electrical discharge machining of this heat-resistant nickel alloy for a wide range of operations.

## 4. Conclusions

The study has led to the following conclusions:

1. The robust regression model for the surface roughness and cut width has been successfully developed for the Wire Electrical Discharge Machining (WEDM) of the heat-resistant nickel alloy.
2. An increase in the height or thickness of the sample leads to a notable increase in surface roughness.
3. The investigation also revealed that increasing pulse on time and the thickness of the workpiece significantly affect the cut width of the specimen.
4. The increase in the height of the sample triggers an adverse impact on the intensity of crack formation on the surface of high-temperature nickel alloys. These cracks, in turn, have detrimental effects on the operational properties of essential products crafted from heat-resistant, next-generation nickel alloys.

## Figures and Tables

**Table 1:** Input parameters for the experiment.

	Pulse on Time, Ton, µs	Pulse off Time, Toff, µs	Workpiece Height, h, mm
Min level	21	21	10
Max level	30	60	15

**Table 2:** Experimentation matrix.

(Trial №)	Input Parameters			Responses	
	Ton, µs	Toff, µs	h, mm	Ra (µm)	Y (mm)
1	21	51	10	1.42	363
2	30	51	10	1.9	333
3	21	60	10	1.09	364
4	30	60	10	1.48	341
5	21	51	15	2.72	380
6	30	51	15	2.5	371
7	21	60	15	2.73	385
8	30	60	15	3.07	372

**Table 3:** ANOVA for surface roughness.

Source	DF	Seq SS	Adj SS	Adj MS	F-Value	*p*-Value
Ton µs	1	0.12251	0.12251	0.12251	2.44	0.216
Toff µs	1	0.00361	0.00361	0.00361	0.07	0.806
h mm	1	3.28961	3.28961	3.28961	65.47	0.004 *
Toff µs × h mm	1	0.22111	0.22111	0.22111	4.40	0.127
Residual error	3	0.15074	0.15074	0.05025		
Total	7	3.78759				

* Significant factors.

**Table 4:** Summary of ANOVA.

Scheme 1	DF	Seq SS	Adj SS	Adj MS	F	*p*
Ton µs	1	703.12	703.12	703.12	121.40	0.002 **
Toff µs	1	28.13	28.12	28.12	4.86	0.115
h mm	1	1431.13	1431.13	1431.13	247.10	0.001 **
Ton µs × h mm	1	120.12	120.12	120.12	20.74	0.020 *
Residual error	3	17.38	17.38	5.79		
Total	7	2299.87				

** Most significant * Significant.

## Data Availability

Not applicable.
